# Reducing Sedentary Time among Older Adults in Assisted Living: Perceptions, Barriers, and Motivators

**DOI:** 10.3390/ijerph17030717

**Published:** 2020-01-22

**Authors:** M. Lauren Voss, J. Paige Pope, Jennifer L. Copeland

**Affiliations:** Department of Kinesiology & Physical Education, University of Lethbridge, 4401 University Drive, Lethbridge, AB T1K 3M4, Canada; lauren.voss@uleth.ca (M.L.V.);

**Keywords:** sedentary behavior, older adults, aging, Social Ecological Model

## Abstract

Older adults accumulate more sedentary time (ST) than any other age group, especially those in assisted living residences (ALRs). Reducing prolonged ST could help maintain function among older adults. However, to develop effective intervention strategies, it is important to understand the factors that influence sedentary behavior. The purpose of this study was to explore perceptions of ST as well as barriers and motivators to reducing ST among older adults in assisted living, in the context of the Social Ecological Model (SEM). Using a qualitative description approach, we sought to learn about participants’ perceptions of sedentary time in their daily lives. Semi-structured focus groups were held at six ALRs with 31 participants (84% women, 83.5 ± 6.5 years). Data were transcribed and coded using an inductive thematic approach. Themes were categorized based on four levels of the SEM: individual, social, physical environment, and organization. Many reported barriers were at the individual level (e.g., lack of motivation, pain, fatigue) while others were associated with the organization or social environment (e.g., safety concerns, lack of activities outside of business hours, and social norms). These findings suggest that there are unique challenges and opportunities to consider when designing ST interventions for assisted living.

## 1. Introduction

Sedentary behavior—sitting or reclining with low energy expenditure while awake—has emerged as a novel risk factor for poor health in the past two decades [1,2]. Prolonged time spent sedentary is associated with an increased risk of obesity, diabetes, cardiovascular disease, and all-cause mortality [3,4,5]. Advancing knowledge on the causes and consequences of excessive sedentary time should be of particular interest to aging researchers and gerontologists, as older adults are the most sedentary age group [4,6], accumulating on average more than 9 h per day of sedentary time [7]. The deleterious effects of sedentary time appear to be greatest among inactive individuals [8,9], which also makes this an important area of study among older adults. Only 4.5% of older Canadians (60–79 years) and 2.5% of older Americans (≥60 years) meet minimum guidelines for physical activity, based on device-measured estimates [6,10]. 

Recent reviews have identified an association between time spent sedentary and several important health outcomes that are particularly relevant to older adults, including functional impairments and reduced mobility [11]. In addition to the total time spent sedentary, the pattern of sedentary time may also be important, as more breaks in sedentary time have been associated with better physical function and cardiorespiratory fitness [12,13]. Therefore, simply breaking up sedentary time with short bouts of standing and light-intensity physical activity could be beneficial to health and function [14]. Given that older adults are sedentary for the majority of their waking hours, and the realization that many of these individuals may be uninterested in or unable to participate in more intense activity, targeting sedentary behavior may be a more realistic approach to increasing daily movement in older adults. A recent international consensus statement [15] identified the need to prioritize research focused on developing effective strategies for reducing sedentary time among older adults. In order to facilitate effective behavior change interventions, it is critical that researchers and practitioners understand older adults’ perception of sedentary behavior, what motivates them to reduce or break up their sedentary time, and common barriers they face when trying to do so.

Recent literature has indicated that older adults frequently identify fatigue, pain, or health problems as barriers to reducing sedentary time [16,17,18]. Another commonly cited barrier is one’s enjoyment of sedentary activities such as watching TV or reading [17,18]. Conversely, motivators for reducing or breaking up sedentary time include improving health and relieving pain and stiffness [16,18]. Completing domestic chores, preparing food, or self-care activities are also frequently cited motivators for breaking up sedentary time [16,17]. Although this research offers valuable insight into the factors that prevent or encourage older adults to limit their sedentary time, all of the aforementioned studies included only community dwelling individuals. 

Population aging, combined with increasing numbers of older adults living with chronic conditions and functional limitations, means that more people are seeking alternative housing options. The World Health Organization forecasts the demand for residential care to quadruple globally by 2050 [19]. Supportive or assisted living environments allow older adults to maintain independence while having access to assistance with activities of daily living and opportunities for social interaction. Assisted living can include a range of services but most have at minimum 24-hour emergency assistance, meal service, and some housekeeping support. Given that domestic chores and food preparation are common motivators for breaking up sedentary time among community dwelling older adults [17], it is not surprising that individuals in assisted-living accumulate less time standing and moving compared to their community dwelling counterparts [20,21]. Residents in care homes in Scotland and Spain reported that the daily routine in their facilities was highly inactive and they felt that they were discouraged by staff from being active due to concerns about safety [22]. Clearly, there are numerous influences on behavior in this type of residential setting, and there are unique challenges and opportunities to consider when designing behavioral interventions for assisted living. 

The Social Ecological Model posits that there are multiple levels of influence on sedentary behavior including individual, social, organizational, and environmental levels [23]. Sedentary behavior is ubiquitous across various domains and perhaps even more so in assisted living. Therefore, it is important to understand whether there are unique barriers or motivators to reducing prolonged sitting that are more relevant to specific levels of influence. Thus, the purpose of this study was to understand perceptions of sedentary behavior, as well as barriers and motivators to reducing prolonged sedentary time among older adults in assisted living. The second purpose was to identify barriers and motivators at different levels of the Social Ecological Model [24], with the goal of informing the development of intervention strategies.

## 2. Materials and Methods 

### 2.1. Study Design and Theoretical Framework

Using a qualitative description approach, we sought to learn about participants’ perceptions of sedentary behavior and their explanations of barriers and motivators to reducing sedentary time in their daily lives. Semi-structured focus groups were used to collect data within a naturalistic framework and analysis was guided by the assumptions of relativism and subjectivism. Consistent with the naturalistic approach, we took the position that a phenomenon is best understood by the meanings participants assign to them [25]. Further, the relativism ontology asserts that reality is subjective [26] and subjectivism is guided by the notion that the world does not exist independent of our knowledge [27]. Thus, subjectivism emphasizes that both the participants and the researcher play a role in constructing knowledge of reality [25]. The study was carried out according the declaration of Helsinki and all study procedures were approved by the University of Alberta Health Research Ethics Board, protocol number 00075411.

### 2.2. Participants

Although “assisted living” is defined differently across and even within regions, for this study, we recruited participants from residences that provide services to older adults in a social model of assisted living that is common in Canada. This means a residence that is a communal dwelling with individual suites, where meals are provided in a group setting, and group activities and social opportunities are available on site. Residences that provide medical care, such as nursing homes or long-term care facilities were excluded and not contacted for recruitment. Participants were purposively recruited from six different assisted living residences in different areas of the same mid-size city in Canada. The smallest residence in this study has capacity for 45 residents and the largest has capacity for 130. Of the six residences that participated, one operates on a private-pay model and five are managed by the same organization which has a government-subsidized funding model. All residences that participated were designated Supportive Living Level 2, which means that all residents are medically and physically stable, not a danger to oneself or others, able to transfer independently, and can self-evacuate in an emergency [28]. 

Volunteer participants were identified through placing flyers and sign-up sheets in common areas of the residence and through discussions with staff who also invited some residents to participate in the study. Inclusion criteria were people at least 65 years of age who had been living in an assisted living residence for at least 6 months. Thirty-one older adults (26 women, 5 men) who ranged from 65 to 97 years of age (Mean: 83.5 ± 6.5 years) participated in seven focus group meetings. Participants ranged in mobility, with 22 able to walk independently with or without an aid and the remaining four using a wheelchair some of the time. All participants were able to transfer independently from seated to standing as this is a requirement to live in the residences that participated in the study.

### 2.3. Focus Groups

Qualitative description methods highlight the importance of studying phenomena in their natural context, and so focus groups were all held on site in a common area or meeting room at each of the six residences. Minimum focus group size was three participants and maximum focus group size was six. Focus groups were conducted using a semi-structured interview guide. All focus groups were conducted between April and May 2018. Prior to beginning the interview, the purpose of the study was explained, and all participants provided written, informed consent. Although first names were used during the focus groups to help build rapport, names were not transcribed, and participants remained anonymous for analysis. For the purposes of presenting the results, the six residences were labelled A-F and the participants in each focus group were assigned a number.

Focus groups were all moderated by one researcher (J.L.C.) and were audio-recorded; the moderator posed open-ended questions and follow-up probes while a research assistant took notes. The interview was broken down into three sections: perceptions of sedentary behavior; barriers to reducing sedentary behavior; and motivators for limiting sedentary behavior (see supplemental data for interview guide). In the first section, participants were asked about their perception of sedentary behavior to gain a comprehensive understanding of how they would define “sedentary behavior” and if they perceived sedentary behavior to be related to health. This allowed participants to explain what “sedentary” meant to them as opposed to telling them what it means, which was important both for building rapport and for providing context to the rest of their ideas and comments. This allowed a better understanding of the meaning participants attributed to the phenomenon of sedentary behavior. After participants gave their own definitions, they were presented with a series of four images, including pictures of older adults engaged in seated activities alone or in groups (watching TV, playing chess, and socializing), then asked about their perception of the activities in those images (e.g., “would you consider these activities as sedentary or not sedentary” and “would you classify these activities as harmful or beneficial”). They were encouraged to explain their answers. The second section of the focus group asked participants to reflect on what motivates them to reduce their sedentary time. Finally, focus group members were asked questions pertaining to the barriers they experience when trying to reduce their sedentary time. 

### 2.4. Analysis

Focus groups were transcribed verbatim and checked for accuracy by a research assistant. Transcripts were read repeatedly by all authors to develop a thorough understanding of the data. Data were analyzed using an inductive thematic approach. Initial coding was conducted independently by two members of the research team (M.L.V. and J.P.P.), who then discussed codes to identify overlap; codes were then sorted into themes. Thereafter, the themes and codes were examined for clarity and consistency by all authors through round table discussions. Once themes and associated quotes had been agreed upon by all authors, each author independently categorized each theme of barriers and motivators according to four levels of the Social Ecological Model: individual, social, physical environment, and organizational. After independent categorization, the authors met to identify and explore any discrepancies; field notes and transcripts were examined again, and final decisions were made in collaboration. The participation of all three researchers in the analysis allowed unique perspectives to be represented in the final themes and categories.

## 3. Results

In general, participants’ descriptions of sedentary behavior had a negative connotation that was associated with being idle. This was illustrated by participant G3, who defined sedentary behavior as *“Just lazy… just sitting.”* Contrary to the formal definition, body posture did not appear to be the defining feature of sedentary behavior for these older adults, as demonstrated by the following quote: *“Sitting down, doing nothing”* (F1). It was consistently communicated that the activity being done is as important to the classification of sedentary behavior as the seated posture itself. Three differentiating factors emerged from participants’ responses, each of which had a positive-negative perception. The first was whether the activity requires cognitive engagement. For example, playing a game like chess was deemed beneficial due to the cognitive stimulation required, as illustrated by this quote from participant G1: *“… yeah, and there’s a game that takes a lot of grey matter. So, I would question whether that one’s truly sedentary*.” In contrast, many participants described watching TV to be a passive activity, and perceived it negatively, as shown by this quote from participant G4: *“Oh, not that one [game], it is social and you’re using your brain. When you’re watching TV someone else is using your brain.”* Although, even TV viewing was considered by some residents to be less passive if it was educational programming or news, as stated by participant F5: *“When you’re watching T.V you’re a… you’re enlightening your horizons, you know for… to learn what’s going in the world”.*

The second important factor that participants highlighted was whether the seated activity was done alone or with others; social interaction was considered positive, even if it was sedentary in nature. For example, when presented with an image of a couple watching TV together, many participants perceived that differently than an image of a person watching TV alone. Participant D3 said succinctly: *“It’s different than watching alone.”* The third factor that participants identified as an important consideration for the classification of sedentary behavior was the duration and frequency of the activity. Overall, participants suggested that some amount of time spent sitting was normal and necessary and that it was only problematic if it was frequent and prolonged, as evidenced by this quote from Participant C3: *“What do I think? It depends on how often they do that is to whether it becomes sedentary or not.”* Participant A4 also made this clear: *“I think if that’s all you’re going to do all day, that is not very good.”* Participant G1 acknowledged that a person’s other movement behaviors matter when considering if sedentary time is a problem: *“Another thing, it depends on what the rest of the life is. I mean, um, without the context of what the whole day is, or in a day, a week, it’s hard to place it because there is a place for sitting down quietly and watching that T.V if you’ve been very active. So, it’s the balance that’s hard to decipher by a picture.”* While passive sedentary time was considered more problematic than cognitively engaging or social sedentary behaviors, these older adults were generally aware that prolonged sedentary time had potentially negative health consequences and that many residents would benefit from reducing or breaking up the amount of time they spent sitting. This was probably summed up best by Participant A2: *“Of course sitting is no good, I know that. No good for my heart, not good for anything”*.

In the next section of the focus groups, we asked participants to discuss their motivators and barriers to reducing time spent sitting. Table 1 shows the nine themes identified in the data that describe motivators for reducing sedentary time. Representative quotes are provided as well as classification of the theme according to the level(s) of influence in the Social Ecological Model. Many of the motivators identified were individual in nature, such as avoiding discomfort and preventing loss of mobility. Several people also talked about using devices to remind them or encourage them to move more. Additionally, there were several motivators that are associated with the social and organizational levels, such as social engagement, companionship, and participating in interesting activities. 

The question about barriers to reducing sedentary time resulted in longer discussions and produced more data. Table 2 lists the 14 themes and representative quotes that describe the barriers to reducing sedentary time that participants identified. As with motivators, many barriers were individual factors, such as fatigue, pain, poor health and lack of mobility. An aging attitude and lack of motivation were also apparent barriers to reducing the time spent in passive sitting. In terms of the physical environment, inclement weather and lack of transportation were identified as barriers to getting out and about, which then increases time spent sitting passively at home. There were also several barriers that seem to be related to organizational structures, such as lack of access to transportation and lack of activities outside of business hours when fewer staff are present. These challenges are compounded by the fact that the organizational setting significantly limits the need for completing activities of daily living, creating further barriers to getting people up and moving. Several participants at one residence also suggested that the small size of their individual suites posed a challenge to incorporating movement into daily life, as there was little space to incorporate moving breaks into otherwise sedentary activities they do on their own, like reading or watching TV. 

## 4. Discussion

Many assisted living sites, including all within this study, provide exercise classes, which are undoubtedly beneficial and should be encouraged. However, they tend to be short bouts of activity on 1–3 days per week, emphasize seated exercises, and are often reported to have low participation rates [21,29]. Targeting sedentary behavior, something older adults engage in for most of the day, may have much broader impact, especially since light intensity activity has been shown to have important benefits, including reducing the risk of cognitive impairments [30]. But in order to develop interventions to reduce sedentary time in assisted living, it is essential to understand the perceptions residents have about sedentary behavior, as well as the factors that could facilitate or hinder their actions towards a less sedentary lifestyle. Sedentary behavior is ubiquitous across leisure, transport, and household domains. So, to produce meaningful, long-term change, organizational and social environments must support and enable alternatives to prolonged sitting. To our knowledge, this is the first study to explore motivators and barriers to reducing sedentary time among older adults in assisted living using a social ecological framework. 

While older adults in this study seemed aware that too much sitting could have health consequences, they did not perceive all sedentary behaviors as “bad”. Participants described a difference between passive or idle sedentary behaviors and activities that were more mentally stimulating or social in nature. This finding is consistent with previous studies of community dwelling older adults, who also suggested that cognitively–engaging or social activities are beneficial to their health and well-being, even when performed while sitting [16,31]. There are limitations in the currently available research that make it difficult to say conclusively whether this assertion is supported by evidence. Regardless, it points to a need to consider this belief when designing interventions. It seems clear that telling people they should avoid card games, crafting, or educational TV programming would not be well-received. In the present study, participants indicated that sedentary pastimes are only problematic if they are pursued for long durations, which suggests that emphasizing breaks in sedentary time as opposed to avoiding sedentary time may be a more successful approach.

Many of the motivators and barriers to reducing sedentary time that we identified are similar to those reported by Greenwood-Hickman et al. [18] among community dwelling older adults who were overweight or obese. It seems that fatigue, pain, health problems, and enjoyment of sedentary activities are individual-level barriers to reducing sedentary time that all older adults face, regardless of their living situation. Similarly, our participants spoke about devices or activity monitors and encouragement from others as important motivators for reducing sedentary time, and these were also described in the study by Greenwood-Hickman et al. [18]. Despite these many similarities, older adults in assisted living face unique barriers to reducing sedentary time.

Participants in this study confirmed that the lack of activities of daily living (ADLs) in assisted living is a barrier to reducing sedentary time, as there is no natural or inherent pressure to move in order maintain a reasonable living situation. We found that this challenge is compounded by aging attitudes, lack of motivation, social norms, and safety concerns, which all promote sitting. This is consistent with the findings of Gine-Garriga et al. [22], who also reported that older adults in residential care spoke of a highly sedentary daily routine with little encouragement to move. The sedentary nature of these residences is supported by evidence that older adults in assisted living accumulate markedly less time standing and walking compared to older adults who live independently [20,21], and studies have shown a rapid decline in function among assisted living residents [32]. Most of the barriers related to the physical environment were not modifiable, except perhaps the problems with access to transportation. Improved access to transportation has the potential to reduce sedentary time by enabling more social participation in the community. Social participation among older adults is associated with less passive sedentary time and more physical activity [33], and so finding ways to support and enable community engagement may help preserve both cognitive and physical function.

Despite the lack of chores and the transportation barriers, other aspects of the residences we visited provide motivation to reduce passive sedentary time, by providing opportunities for social engagement and interesting activities. Furthermore, the communal food service was frequently cited as the most common reason people would get up and walk to the main floor of the residence. Meals or coffee were mentioned by people at every focus group meeting as a motivator to leave their individual suites and thus break up sedentary time. While this could be considered an individual-level determinant of behavior, there was an overarching sense that this was highly influenced by the social aspect of the activity. As stated by Participant E2: *“You go down. There’s always a lot of people around so you go down, get a coffee and hang out, visit”*. As seen in Table 1, organized activities were also an important motivator for many people to leave their rooms and move around the facility or go out. This suggests that planning more activities and incorporating “moving breaks” into organized sedentary activities may be a way to reduce the sedentary time in the daily routine. This is supported by the fact that many residents commented on a lack of organized activities on evenings and weekends as a barrier. The quotes shown in Table 2 related to the lack of weekend activities illustrate the importance of organized activities in encouraging residents reduce passive, isolated sedentary time. Motivation and pleasure are key considerations to changing movement behaviors among residents of assisted living [34] which suggests that encouraging and supporting residents to independently initiate social activities or events in the evenings or weekends could be a beneficial strategy. Future research should explore this possibility. 

Research on sedentary behavior to date has generally focused on community dwelling and younger adults and many studies have reported findings similar to the ones presented here. Nooijen et al. [35] found that the key reported barriers to reducing office-based sitting were individual habits and a belief that standing was physically taxing. They also reported that organizational factors, like standing meetings and reminders to take a break from sitting, helped them to limit sedentary time. A thematic synthesis of qualitative studies with community dwelling adults [36] identified factors at all levels of the Social Ecological Model that influenced sedentary time, including: enjoyment of sedentary activities, weather, and social support. The present findings confirm that many of these same factors are also relevant to older adults in assisted living and should be considered when trying to alter sedentary behavior patterns.

Despite some similarities with previous studies, our results provide new insight into motivators and barriers that are unique to an assisted living environment. For example, while Chastin et al. [16] found that activities of daily living and household chores were a key motive for reducing sedentary time in community dwelling older adults, in assisted living the absence of domestic chores was identified as a barrier. To our knowledge, only one other study has examined barriers or motivators to reducing sedentary time within an assisted living environment [22] although the results focused more on the benefits of being physically active or exercising rather than the benefits of breaking up prolonged sitting. Participants in that study [22] offered suggestions that were consistent with our findings, including: support from family and staff; giving residents a purposeful reason to move (i.e. increase ADLs); and more frequent reminders to residents to break up their passive sedentary time. The presence of staff and organized social activities where people live are unique attributes of assisted living residences; this creates opportunities to change behavior that aren’t readily available to those who live independently. 

The results of this study highlight the interaction between the levels of the Social Ecological Model. For example, the organized activities are integrated at the individual, social, and organizational level. At the organizational level, these activities and events are the responsibility of the staff who plan and deliver them. These activities then serve as an individual motivator for people who enjoy them, and they also influence residents at the social level by motivating residents to engage in the activities specifically for social interaction. However, staff constraints can also act as a barrier to residents engaging in these types of activities at certain times, like evenings and weekends, and may result in a lack of motivation to interrupt prolonged sedentary time. The importance of staff engagement was clear, and some interventions within assisted living have started to implement organizational strategies rather than focusing on individual behavior change [37]. Whether or not that approach is successful remains to be determined. Similar strategies have been used with some success in workplace research [38]. Engaging key management positions and altering social elements within the office environment have both been shown to be important for facilitating individual behavior change; we posit that a similar approach is needed in assisted living. Encouragement from family members and medical professionals to reduce sedentary time also emerged as an important motivator, thus, educating those individuals on the benefits of reducing sedentary time could be another important strategy to help limit prolonged sedentary time among older adults. 

There are some limitations to this study. The participants were predominantly women and only four used a wheelchair for mobility support. It is possible that men and individuals with more restricted mobility have unique perceptions of sedentary time that were not fully captured in this study. We also did not assess the sedentary time of participants; people with more or less daily sedentary time may perceive the behavior differently. Further, this study utilized a qualitative description approach which acknowledges that both the participants and the researcher play a role in constructing knowledge. The researchers were all women who are younger than the participants, do not reside in or work at an assisted living facility, and have considerable knowledge of the sedentary behavior literature. It is possible that if the researchers were different genders or had different backgrounds, participants would have provided different responses or the results would have been interpreted differently. Finally, this study only included assisted living residences with a similar model of care in one city; results may not be generalizable to all types of assisted living or regions.

## 5. Conclusions

This study demonstrated that individual characteristics and beliefs are not the only barriers and motivators to reducing sedentary time and that there are unique factors in assisted living residences that influence sedentary behavior. The results suggest that reducing sedentary time in assisted living could be achieved without high-cost programs or additional staff, by capitalizing on the social opportunities that already exist in this particular model of assisted living. Using the Social Ecological Model as a framework, strategies should target a shift in habits at the individual, social, and organizational levels. Strategies at each level must focus on providing education and encouragement, increasing motivation, and providing interesting and enjoyable alternatives to prolonged periods of passive sedentary behavior.

## Figures and Tables

**Table 1:** Themes related to motivators for reducing sedentary time among older adults in assisted living, and associated level(s) of the Social Ecological Model (SEM).

Theme	Quote	SEM Level
Devices	G2: “Well I use a timer… If I am going, um, say like even if I’m tired in the afternoon, if I want to have a rest, I’ll set a timer for 20 min/half an hour, put my feet up in my armchair and just, you know? Relax, and then … time’s up. Better get moving again.” D1: “…band that shows my steps, you know? …And it made me go, I wanted to see how many steps I get.” F1: “I think it [pedometer] does, it gives you something- a reason. You think “Oh did I move or didn’t I”?”	Individual
Avoid Discomfort	A6: “The more you sit, the sorer you get.” D2: “And I do the stairs. Actually, I can get up from the dining room and I feel stiff and I walk up the stairs and it helps…” A2: “You know I have a small room, and yesterday only I walked 20 min. In my room with my walk, and I’m watching the time, and then when I’m finished my knees feel a bit looser.”	Individual
Prevent Loss of Mobility	C3: “…there’s still another level to assisted living, and you don’t want to move in that direction, so you try and keep yourself going. And [AC name] here provides all kinds of programs but it’s still your choice as to whether you want to do it or not. And uh, and I think people need to choose to do that kind of stuff—if they don’t, they’re just going to slip away.” E2: “I have arthritis and I know the worst thing you can do with arthritis is to not move, so I... that’s why I do it” G1: “Although I see people whose health is far from good and they work harder at staying active than moving up and down the halls doing what they can do, and they are healthier for it than those that don’t participate.”	Individual
Food	D3: “That’s our biggest motivation, going for meals and going down for coffee.” B3: “Well, I don’t get up unless I just have to seems like. But if I do get up, you know to go for meals and that sort of thing.”	Individual, Social
Sense of Obligation	C3: “Been sitting for an hour and get the hell off your ass, you know?” G4: “Oh yeah. Um, yeah, I can do—sometimes I think ‘I got to get up and walk around or go out and about’, something like that.	Individual
Identity	E3: “Well it’s just by nature I guess because I always feel like I should be doing something instead of just idle.” G1: “I’ve never been somebody that wasn’t active. I think maybe I don’t know how to not be active.”	Individual
Encouragement	D1: “Oh yeah, the doctor says, ‘every half hour, get up and move.’” A6: “Well, I’m going to tell you something. This, my daughter’s a nurse and she says ‘mum, walk’. She says ‘you’re gonna be in a wheelchair’. I said “’well sometimes my back’s too sore’. ‘Walk anyways, it’ll help your back.’ And you know what? It does.” C1: “I’m motivated by my son…He phones me…’Are you getting up? Are you getting up and moving around every so often?’...”	Social
Social Engagement	G2: “Like, uh, I come down for supper every night and just thoroughly enjoy it because that’s when I get to see everybody and visit with them” D2: “You go down. There’s always a lot of people around so you go down, get a coffee and hang out, visit.” G4: “…you have to walk from your room, which is quite a distance, downstairs and back up again. So, you’re still getting some exercise out of it and also you’re getting a great deal of sociability”	Social, Organizational
Companionship	F6: “I go for the buddy system, you know? And that’s another thing that a lot of people, older people, they don’t go, but if they had a friend that would go with them... If you got just one person that can give you a little challenge, you know? Or you can work out with that person, male or female I don’t care.”	Social
Interesting Activities	D2: “… [AC name] gives us things that, like the um…anyway, check a sheet and we went to four floors to find the clues…A scavenger hunt. We don’t pick it up, we just find the clues. That’s a lot of walking because it’s four floors. And… by the time we are finished she says, we’re just doing it to give you the exercise, which we know of course.” C2: “And if there’s an activity that we could do things, I’m there” C3: “I enjoy going out, I enjoy just, uh, any of the trips that we have here. I enjoy doing those.”	Individual, Organizational

AC: activity coordinator.

**Table 2:** Themes related to barriers to reducing sedentary time among older adults in assisted living, and associated level(s) of the Social Ecological Model (SEM).

Theme	Quote	SEM Level
Aging Attitude	B3: “Well I enjoy it [T.V], and when you get my age you can’t get out to do anything else.” G3: “Well, I don’t know. I mean what can old people do?”	Individual
Too Much Effort	D3: “It’s very difficult because…because sitting is so easy.” D1: “Well what do you do in a place like this, when you’re not doing…you know. I’m 97. I don’t, you know, I don’t get into everything like I used to…I just don’t do it. So, when you come back to your room, you sit.” F1: “Course not, it’s too hard to get up.”	Individual
Enjoyment of Sedentary Behaviors	D4: “I enjoy my computer and my TV, of course.” G2: “And now I read all the time, so I guess that’s another reason to say that it’s an absolute joy to be able to read. “ B3: “Well I enjoy it [TV], and when you get my age you can’t get out to do anything else.”	Individual
Fatigue	G3: “I don’t know, I just—during the day I keep busy, I’m always going someplace and doing something, but in the evening, I’m sitting. Because after so many hours of being out and walking, I need to sit down and rest.” D4: “I find it hard when I’m walking, in the store or do something, or visiting. I’ve got to listen to my body…and I want to go to Walmart, and I say no, you better go home.”	Individual
Health	B1: “Well I have a back problem, it’s awful when you have to … to straighten up and do things.” D4: “And, I walk around the floors, but I can’t do too much walking with my heart condition.”	Individual
Mobility	E2: “Well, I wish I’d be able to walk without help…that’s the biggest thing.” B3: “Our nurse, yesterday or so, she said ‘you should get more exercise, you’re using that wheelchair so much you should go back to your walker’. And I agreed with her but, but I just can’t do it. I walk out for a meal and then I can’t get up out of the chair. I have to have help getting up.”	Individual
Pain	F1: “You’d stand up more if it didn’t hurt.” C3: “I think that your body is deteriorating. My bottom line is always we are living too long, too long. I mean at 80 years old I’ve been standing upright for at least 79 of those years. You know and I just—I don’t know why we’re living so long, and I don’t you could find anyone in here that doesn’t have discomfort of some sort, and you just- “	Individual
Lack of Motivation	D3: “And I don’t think, most of us probably don’t move enough. That’s probably the weakness. Is as you get older, it’s harder to motivate you to move.” C3: “I see probably motivation begins with yourself … and your attitude. And, uh, you can’t force people to do things don’t want to.” E1: “And some days you think “oh I should move myself”, and then other days, you really don’t care”	Individual
Fear of Falling	F1: “You can’t stand up, that would—you’d be picking us all up off the floor.” D3: “And most of us, I would hazard a guess, are afraid of falling.” C3: “Because they [staff] don’t want people falling and cracking their hips…”	Individual, Organizational
Social Norms	A6: “Because I need all of this to get going, and um, I would just love to see more people getting involved. But people just sit in their rooms or their, you know? And they don’t do anything.” G4: “There’s a whole group that entertain themselves in their rooms…so they’re missing the sociability” A2: “…but I have no one to walk down the stairs with.”	Social, Organizational
Weather	C1: “This winter has been really bad for me because I’m an outdoorsy person, and it was really hard to even get in the car and go anywhere because the weather was bad all winter.” E1: “Believe me, there are some beautiful things in the winter—like you see cardinals down in the wetlands and it’s beautiful to see them. There are some beautiful sights but you, you just can’t do it in the winter—not walking.”	Physical Environment
Transportation	A5: “I believe I have an advantage over a lot of people in here. If [name] and I really get tired of sitting in here, we jump in the van and take off.” G1: “Something I think can make it easier for people to remain motivated, um, especially in our age group were people can’t drive any longer, you have to be physically able to go out there and pick up a city bus, or you have to be financially in a position where you qualify for Access-A-Ride, and this annoys me, that Access-A-Ride. … I believe it should be available at spots like this.” A6: “Yeah, the transportation is a problem.”	Physical Environment, Organizational
Lack of ADLs	D3: “And you know, we’re all old, old here. But until we moved in here, we did a lot of activity just living, looking after where we were living…” A5: “Well I don’t get up unless I just have to, seems like … But all I do anymore is eat and sleep and that’s about it.”	Organizational
Lack of Activities (Weekends and Evenings)	A5: “Well here’s another thing like say our weekends around it’s very dull, there’s no activities whatsoever.” A6: “What I mean by that is after supper I just sort of sit in my big chair and watch T.V. No good. No good whatsoever. And, um, I’m going to playing some cards after our meeting and that’s fun and, um I find that weekends here are not very enjoyable, nothing to do.” F6: “But Saturday and Sunday we- feels like a morgue…Should have something on weekends. We have nobody here on weekends.”	Organizational

AC: activity coordinator.

## Supplementary Materials

The following are available online at https://www.mdpi.com/1660-4601/17/3/717/s1, Focus Group Guide.
