# Supplementary material for: Reducing Sedentary Time among Older Adults in Assisted Living: Perceptions, Barriers, and Motivators
Source: Int J Environ Res Public Health. 2020 Jan 22;17(3):717. doi: 10.3390/ijerph17030717 (PMC7037380; doi:10.3390/ijerph17030717)
Supplement: Supplementary file 1 [file ijerph-17-00717-s001.pdf]

Focus Group Guide

**Start with consent process and introductions; name placards**

**Introduction:**

*Thank you for coming today and talking to us. Your input is so important for our project and we really appreciate your help! We are doing research on healthy ageing and in particular we are interested in talking about sedentary behavior. We want to know what you consider to be sedentary, and what kind of things might encourage or motivate you to be less sedentary in your daily life. There are no right or wrong answers, but rather your honest opinions are important to us.*

| Question | Purpose of question(s) |
| --- | --- |
| 1. What does the word "sedentary" mean to you? (probe: how would you explain sedentary behavior to someone else?). | To determine how residents perceive sedentary behavior; to determine their awareness of the health implications of sedentary behaviour. |
| 2. Can you give examples of activities that you would label as sedentary?<br>Probe - What are some examples of sedentary behaviors that people do: outside<br>Inside<br>For fun<br>As a hobby<br>When they are being social | |
| 3. If you look at these images, can you tell us if you think they are (1) sedentary/not sedentary, and (2) "good for you" or "bad for you", and why? Probe: maybe good or bad for your physical or mental health, or for your social life?<br>Probe: how do these images make you feel?) | |
| 4. Do you think spending time in these activities is beneficial or harmful? Or neither?<br>Probe: Why do you think these activities are beneficial/harmful/neither<br>What do you think are the benefits/drawbacks of these activities? | |
| 5. Do you intentionally engage in or avoid these sedentary activities?<br>Probe: How often do you do these activities?<br>Probe: How long do you typically sit before standing or moving?<br>Probe: Do you ever purposely break up your sitting time with standing or moving? | |

<table>
<tr><td colspan="2">DEFINE SEDENTARY BEHAVIOUR<br>Transition: This next series of questions is going to ask you for more ideas about sedentary activities and what you think about them. We want to know what motivates you or prevents you from doing different activities.</td></tr>
<tr><td>5. Do you think people you know spend too much time sitting or lying down?<br>6. How much time do you think you spend sitting in a day?</td><td>To determine awareness of the pervasiveness sedentary behaviour.</td></tr>
<tr><td>7. What might motivate you to move out of your chair more often during the day?<br>Probe: What do you do to sit less/break up your sitting time?<br>Probe: what are you willing to do to sit less/break up your sitting time</td><td>To identify motivators for reducing sedentary behaviour.</td></tr>
<tr><td>8. If you wanted to sit less, what kinds of things might prevent you (Probe: or other people) from sitting less during the day?</td><td rowspan="2">To identify barriers to reducing sitting time.</td></tr>
<tr><td>9. What kinds of things might prevent you from standing up and moving around more frequently?</td></tr>
</table>
